# A Bayesian Network–Based Browsing Model for Patients Seeking Radiology-Related Information on Hospital Websites: Development and Usability Study

**DOI:** 10.2196/14794

**Published:** 2021-01-19

**Authors:** Ryusuke Suzuki, Teppei Suzuki, Shintaro Tsuji, Kensuke Fujiwara, Hiroko Yamashina, Akira Endoh, Katsuhiko Ogasawara

**Affiliations:** 1 Graduate School of Health Sciences Hokkaido University Sapporo Japan; 2 Iwamizawa Campus Hokkaido University of Education Iwamizawa Japan; 3 Graduate School of Commerce Otaru University of Commerce Otaru Japan; 4 Department of Medical Informatics Hokkaido University Hospital Sapporo Japan

**Keywords:** web marketing, internet, hospitals, radiology, information-seeking behavior

## Abstract

**Background:**

An increasing number of people are visiting hospital websites to seek better services and treatments compared to the past. It is therefore important for hospitals to develop websites to meet the needs of their patients. However, few studies have investigated whether and how the current hospital websites meet the patient’s needs. Above all, in radiation departments, it may be difficult for patients to obtain the desired information regarding modality and diagnosis because such information is subdivided when described on a website.

**Objective:**

The purpose of this study is to suggest a hospital website search behavior model by analyzing the browsing behavior model using a Bayesian network from the perspective of one-to-one marketing.

**Methods:**

First, we followed the website access log of Hokkaido University Hospital, which was collected from September 1, 2016, to August 31, 2017, and analyzed the access log using Google Analytics. Second, we specified the access records related to radiology from visitor browsing pages and keywords. Third, using these resources, we structured 3 Bayesian network models based on specific patient needs: radiotherapy, nuclear medicine examination, and radiological diagnosis. Analyzing each model, this study considered why some visitors could not reach their desired page and improvements to meet the needs of visitors seeking radiology-related information.

**Results:**

The radiotherapy model showed that 74% (67/90) of the target visitors could reach their requested page, but only 2% (2/90) could reach the Center page where inspection information, one of their requested pages, is posted. By analyzing the behavior of the visitors, we clarified that connecting with the radiotherapy and radiological diagnosis pages is useful for increasing the proportion of patients reaching their requested page.

**Conclusions:**

We proposed solutions for patient web-browsing accessibility based on a Bayesian network. Further analysis is necessary to verify the accuracy of the proposed model in comparison to other models. It is expected that information provided on hospital websites will be improved using this method.

## Introduction

### Hospital Web Marketing in Japan

For patients to choose a preferred hospital, information transmission using a hospital website is important because the external environment is constantly changing, owing to improvements to the internet and new competitive relationships as a result of the declining trend of patients [1,2]. It is assumed that there are 2 major reasons why information on websites has become increasingly important for hospital management. First, patients can choose a hospital based on their personal preferences. The “paternalism model,” where physicians make decisions, was previously the mainstream; however, patients have shifted to an “informed decision model,” where they make decisions themselves [3]. A previous study conducted by the Japanese Ministry of Health, Labor, and Welfare revealed that the percentage of those who responded that “I have obtained information” for the item “Is information usually obtained when you visit the hospital?” increased by 30% compared to 2011 [4]. Second, the popularization of smartphones among all age groups has led to significant lifestyle changes [5,6]. Patients can select a hospital based on the information available on a website. According to the National Federation of Health Insurance Societies [7], the information from a hospital website has the largest impact on hospital selection. The internet serves as an important source of medical information [8]. A study showed that “hospital websites” and “information websites about hospitals”’ account for a high proportion of the available credible information.

In previous research targeting hospitals in Japan, the introduction of marketing departments in hospitals has recently been proposed [9]. However, medical advertising guidelines make it difficult for hospitals to advertise as a company [10]. In today’s society, with advances in the internet, it is expected that the proportion of individuals who choose to seek information from such sources when deciding on a hospital for an examination or treatment will continue to increase, despite the strict regulations. Therefore, hospitals need their own web marketing methods.

### Improve Information Provision on Hospital Websites

Through recent marketing, CRM, one-to-one marketing, and personalization aimed at increasing patient satisfaction and loyalty by reaching individual consumers through the spread of the internet and intensified competition are attracting attention [11,12]. It is therefore desirable to transmit appropriate information to the websites according to the needs of each visitor. For this reason, to improve information provisioning on websites, it is common to analyze the current state of websites using quantitative data such as access logs. As advertising regulations were adopted on websites in Japan, the study focused on whether the information posted on websites followed the regulations [13,14]. In the United States, there are weaker advertising regulations on websites than Japan; a study evaluated the accessibility of websites to provide high-fidelity information from the viewpoint of public health [15]. Few have focused on the browsing behaviors of visitors on hospital websites. Suzuki et al [16] attempted to develop a method for creating browsing behavior models and assessing the intentions of patients on hospital websites. However, no models have been proposed that allow medical staff to intuitively understand the browsing behavior of patients and propose improvements to meet the needs of visitors seeking radiology-related information. Therefore, we focused on a Bayesian network that can easily understand variable relationships.

Owing to the market trends, we decided to analyze not all visitors but specific visitors. Our research target, Hokkaido University Hospital, is one of the largest hospitals in Japan with 944 beds. Furthermore, Hokkaido University Hospital has one of the 17 proton therapy facilities in the country and therefore provides advanced medical care in the field of radiotherapy [17]. It is possible that there was a certain need at this hospital to provide information in response to citizen requests for the transmission of radiology-related information. In addition, after the Great East Japan Earthquake (March 11, 2011), several individuals have become interested in radiology-related information. Some studies have reported that few people have sufficient knowledge of radiology, and many people are concerned about exposure to radiation [18,19]. Owing to these situations, several patients have sought information on the safety and effects of radiotherapy and radiological diagnosis. Furthermore, there are various types of radiological diagnoses, such as radiography, computed tomography, and magnetic resonance imaging, which make it difficult for patients to understand each type of examination. This study conducted a behavioral analysis focusing on visitors seeking radiology-related information.

The purpose of this study is to propose a hospital website search behavior model in which medical staff can easily understand the browsing scenario by analyzing the browsing behavior of visitors who are looking for radiology-related information based on the results of a Bayesian network from the perspective of one-to-one marketing.

## Methods

### Scheme

**Figure 1:**
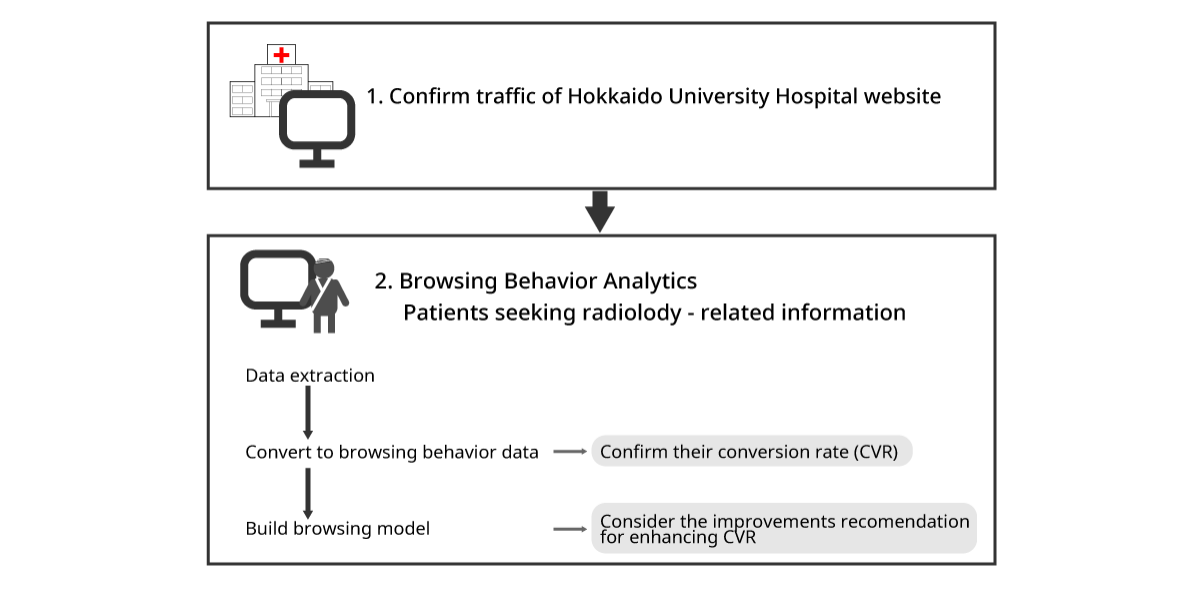
Research scheme.

**Figure 2:**
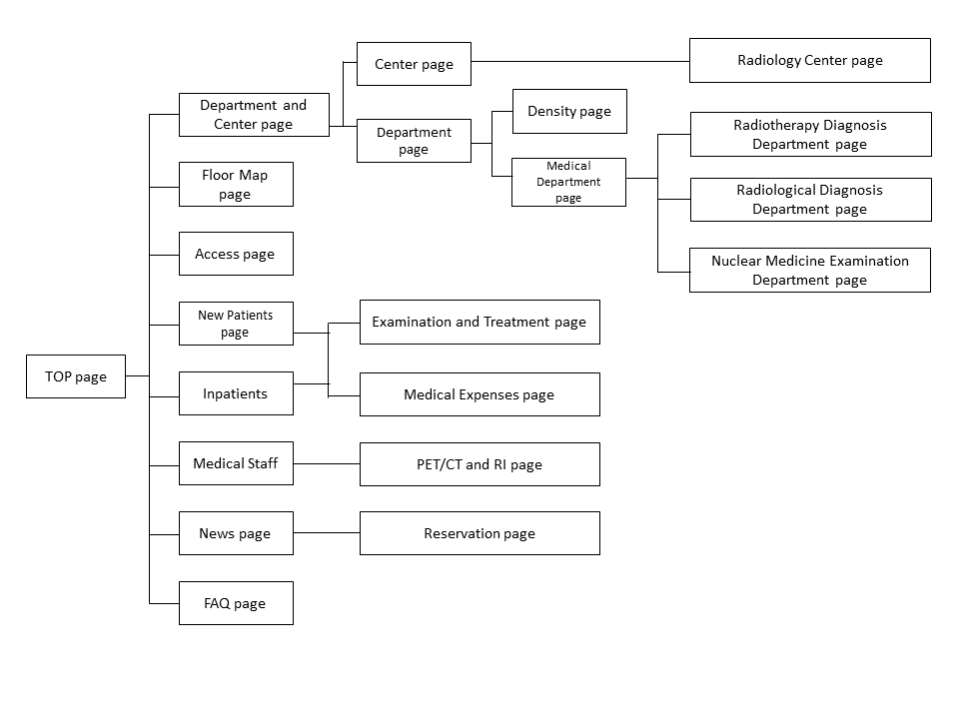
Abstract of target website link about radiology-related information. PRT-CT: positron emission tomography-computed tomography; RI: radio isotope.

**Figure 3:**
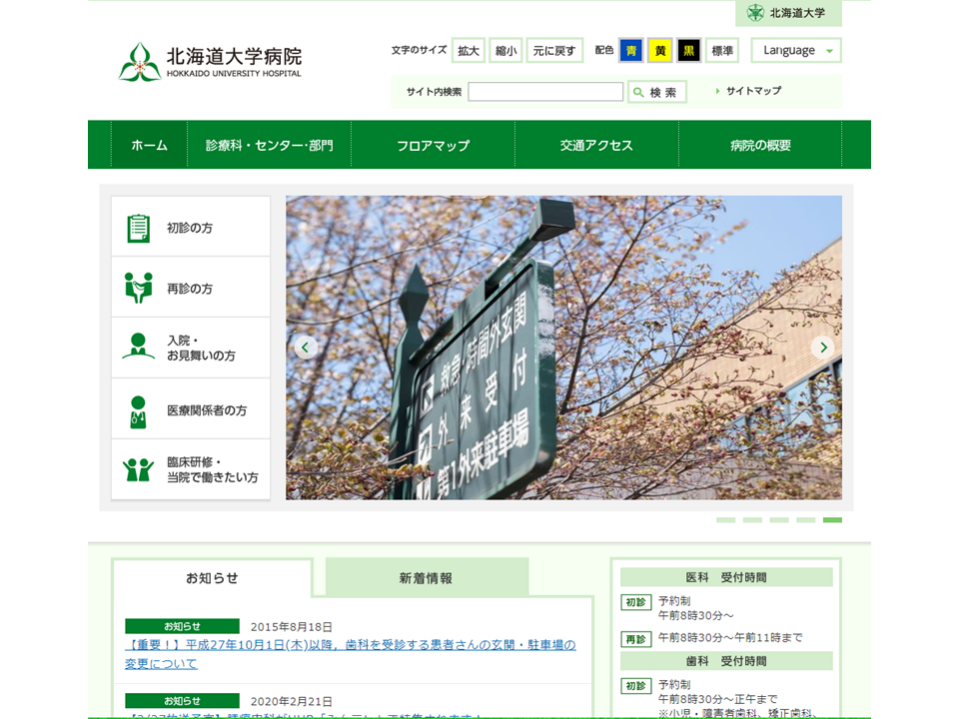
TOP page of Hokkaido University Hospital website (personal computer edition).

A flowchart of our research is shown in Figure 1. The page that we believe the target wants to browse was defined as the “request page (RP).” In addition, a target reaching the requested page was defined as a “conversion,” and the ratio of conversion as the “conversion rate (CVR),” as shown in 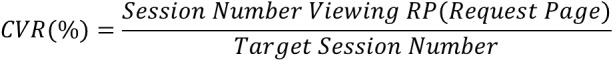.We attempted to enhance the CVR using a Bayesian network with an access log. We used the website data access log of the Hokkaido University Hospital website (Figures 2 and 3), which has 208 pages. Our research focused on the patients seeking radiology-related information at Hokkaido University Hospital as the target visitors. 

### Traffic Confirmation

First, we collected the website access log of Hokkaido University Hospital from September 1, 2016, to August 31, 2017. Knowing the purpose and needs of the visitors is useful for improving a website; however, this information usually cannot be observed, and it is necessary to understand this information through an analysis of the data access log. This research analyzed the access log using Google Analytics. Google Analytics is a tool that allows users to visualize the access status without a server that even medical clerks can easily handle. Google Analytics has been widely used in public institutions, including hospitals [16,20]. We used “page view,” which shows the number of pages viewed by visitors, “session,” which shows the number of times any visitor accessed the website, and “page depth,” which shows the number of pages viewed in a single session.

#### Data Extraction

First, we acquired data from the access log by designating 8 indicators using Analytics Edge, which is an add-in service of Google Analytics. The following metrics were collected from the user’s http request: 1, page title; 2, retrieval keyword by visitor; 3, municipalities of visitors; 4, browsing date by visitor; 5, network domain of the visitor; 6, page depth; 7, operation system of the visitor; and 8, page views. Although Google Analytics issues a “Client ID,” which identifies the user, the website administrator cannot obtain the ID. Thus, the administrator cannot identify the individual user data from the raw data. Therefore, the behavior data of each user was identified by generating a pseudo user ID from indicators 2 to 7, which led to the identification of the user. We used only indicators 3 to 7 to identify the user. We extracted data for 870 sessions using 32 radiology-related keywords that the target would use as a retrieval keyword, as shown in Multimedia Appendix 1. This selection was made by a specialist in the field of radiology. The exclusion conditions in the data extraction used were as follows: we excluded the records of the session with a page depth of 1 because of the difficulty in interpreting the intention to visit [21]. To acquire patient data, we excluded the following types of data used in retrieval keywords: recruitment-related words (eg, touring and recruitment), technical terms (eg, academic society and doctor names), and words related to needs other than treatment and examination. We also excluded data with “.ac.jp” at the end of the network domain, which is called the second-level domain.

Second, we classified the extracted sessions into 3 types of needs using retrieval keywords: 1, sought radiotherapy information; 2, sought information on nuclear medicine examination; and 3, sought information on radiological diagnosis. The judgment of the 3 classifications was based on an interpretation of indicator 1: retrieval keywords by a specialist in radiology.

Third, the RP for the 3 groups were selected, as shown in Table 1. However, the RP was an estimate because we could not know for certain what information was needed. Thus, we chose the RP in order of high scores for “Explanation of flow of examination,” “Request/notes,” “Explanation of technical terms,” and “Explanation of equipment” in reference to the previous study, for the 3 groups from the target website [22]. Table 2 shows the evaluation criteria. The Center Navigation page, that is, the link source of the Radiology Center page, was set as RP1. Because the Radiology page was an external link, we could not examine the access log.

**Table 1:** Request pages in each model.

Group	Request pages			
	1	2	3	4
Radiotherapy	Center Navigation	Radiotherapy Department	—^a^	—
Nuclear medicine examination	Center Navigation	Nuclear Medicine Examination Department	Examination and Treatment	PRT-CT^b^ and Nuclear Medicine
Radiological diagnosis	Center Navigation	Radiological Diagnosis Department	Examination and Treatment	—

^a^The request pages (RPs) for 3 groups were set by a specialist in radiology. The lower rank RP in a certain group was not applicable in our RP selection.

^b^PRT-CT: positron emission tomography-computed tomography.

**Table 2:** Criterion for page evaluation.

Assessment and condition		Score
**Equipment description**		
	Statement	2
	No statement	1
**Doctor introduction**		
	Statement	2
	No statement	1
**Records of previous diagnoses**		
	Statement	2
	No statement	1
**Flow of examination**		
	Statement (image and text)	3
	Statement (only text)	2
	No statement	1
**Precautions**		
	Statement	2
	No statement	1
**Terminology**		
	Statement (including meaning of the term)	2
	Statement	1

Fourth, we converted the extracted access log into behavioral data. These behavioral data show whether each visitor browsed each page. After calculating CVR, these data were applied to the Bayesian network.

#### Browsing Models

**Figure 4:**
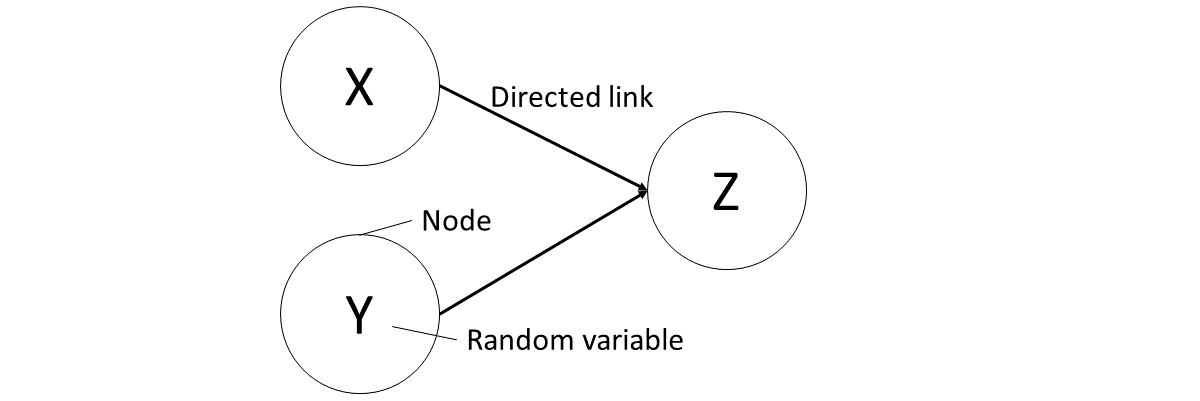
Bayesian network outline.

Behavioral models were built based on the Bayesian network model, using BAYOLINK version 7.0.1. A Bayesian network is a probabilistic model that expresses the qualitative dependence among multiple random variables using a graph structure and expresses the quantitative relationship between individual variables based on their conditional probability. Variables are nodes, and a linked node is referred to as a “child node,” and a link source node is referred to as a “parent node,” as shown in Figure 4. In addition, Bayesian network can be used to determine the probability distributions of child node by observing the parent node. When using statistical models in marketing, it is crucial to determine whether the model is useful for decision making. Bayesian networks can express relationships between explanatory variables and even model the entire structure [23]. Although previous studies on predicting website-browsing behavior used Markov models and n-grams, such approaches were based on the premise that recent page access records have a significant influence on future page accesses [24,25]. In our models, the input value was binary for the browsing or nonbrowsing of each page for the visitors based on the premise of prior research. In other words, whether the page was browsed was represented as a node of the network. To determine the structure of the network and the parameters, we adopted Akaike’s information criterion as the standard for selecting the models and the greedy search algorithm as the standard for deciding on the construction of the models. Their criterion shows adaptability to data and simplicity. The greedy search algorithm is a method of dividing the problem element into a plurality of partial problems, independently evaluating each parent combination with the highest evaluation value. In this study, all nodes were designated as parent node candidates. In addition, to show the models briefly, we built each of the models by setting pages with a small number of views as “Other pages.” In our models, all the variables were binary and indicated whether browsing was performed. The joint probability indicates the probability of an arbitrary behavior being observed, and the conditional probability distribution indicates the distribution of the browsing status of the target pages considering that of a certain page. Therefore, our models show the cause-and-effect relationship between the browsing pages of the target visitor. For example, in a model in which the child node is the treatment page and the parent node is the department page, we interpret this to mean that the browsing probability for the treatment page is affected by the browsing probability for the department page. A method was then necessary to judge the strength of the influence.

After structuring the browsing model, the sensitivity analysis was performed to determine the influence of other variables in the model on any variable and searched for pages that affected the browsing probability of the RP using mutual information. Mutual information content represents a measure of the independence of 2 random variables. Referring to the results of the sensitivity analysis, hypothetical experiments called a probability inference were conducted to determine the probability distribution of any other variable when observing certain variables and predicted the resulting variable, that is, the browsing probability of the RP.

Finally, some improvements were discussed to increase the browsing probability of the RP and meet the needs indicated from the models.

## Results

### Traffic Summary of the Target Website

There were 2,521,279 sessions during the study period, as presented in Table 3. Access to the department pages was concentrated next to the TOP page, as listed in Table 4. After data extraction, a behavior analysis was conducted for a total of 90 sessions, including 35 sessions for the radiotherapy interest group, 35 sessions for the nuclear medicine examination interest group, and 20 sessions for the radiological diagnosis interest group. The CVR for the 3 groups is presented in Table 5. In our study, 74% (67/90) of the targets could reach the RP. 

**Table 3:** Access during the study period.

Indication	Values
Page views, n	2,521,279
Sessions, n	663,213
Users, n	381,307
Average pages viewed per sessions	3.8
Average session time (second)	146

**Table 4:** Access rate of main pages and radiology-related pages.

Page title	Page views, n (%)
TOP	362,785 (14.39)
Department Navigation	187,558 (7.44)
Medical Department Navigation	153,454 (6.09)
Department and Center Navigation	123,462 (4.90)
Access	99,806 (3.96)
Center Navigation	28,190 (1.12)
Radiotherapy Department	7757 (0.31)
Radiological Diagnosis Department	4839 (0.19)
Nuclear Medicine Examination Department	4651 (0.18)
Examination and Treatment	2407 (0.10)

**Table 5:** Accessibility to request pages in each model.

Group	CVR^a^ (%)				
	RP^b^1 (%)	RP2 (%)	RP3 (%)	RP4 (%)	Total (%)
Radiotherapy	2.9	80	—^c^	—	80
Nuclear medicine examination	2.9	31	9.0	57	83
Radiological diagnosis	0	45	5.0	—	50

^a^CVR: conversion rate.

^b^RP: request page.

^c^The lower rank RP in certain groups was not set in our judgement. Therefore, certain CVRs were treated as not applicable.

### Radiotherapy Interest Group

**Figure 5:**
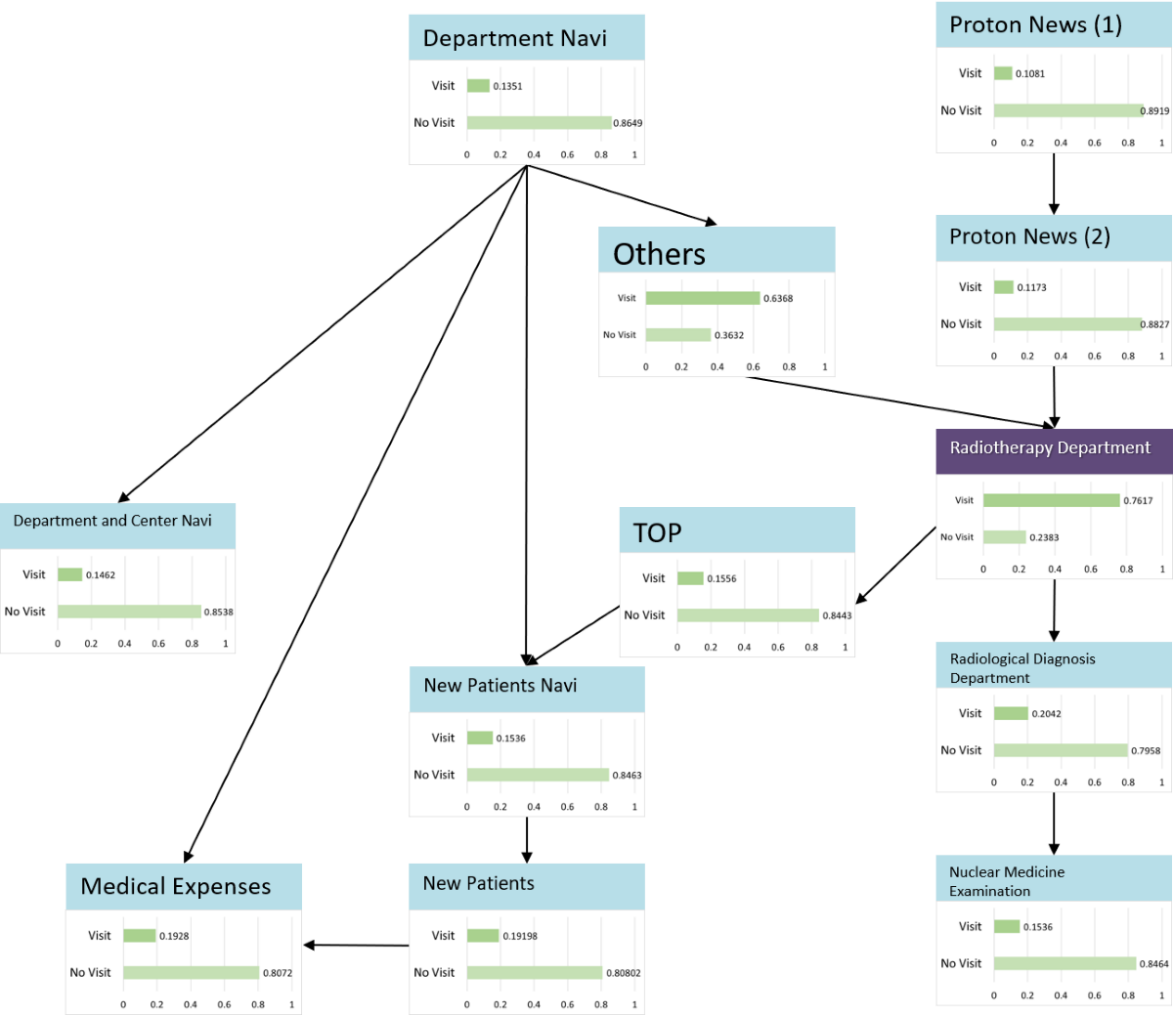
Radiotherapy model.

**Figure 6:**
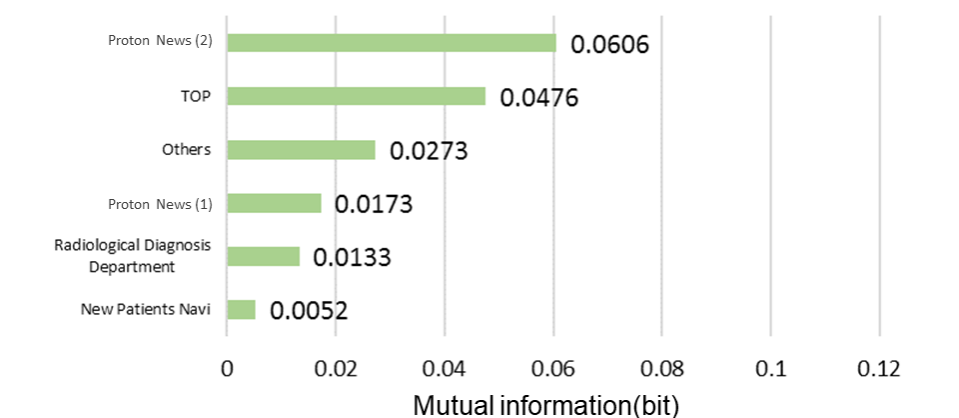
Sensitivity analysis (radiotherapy interest group).

**Figure 7:**
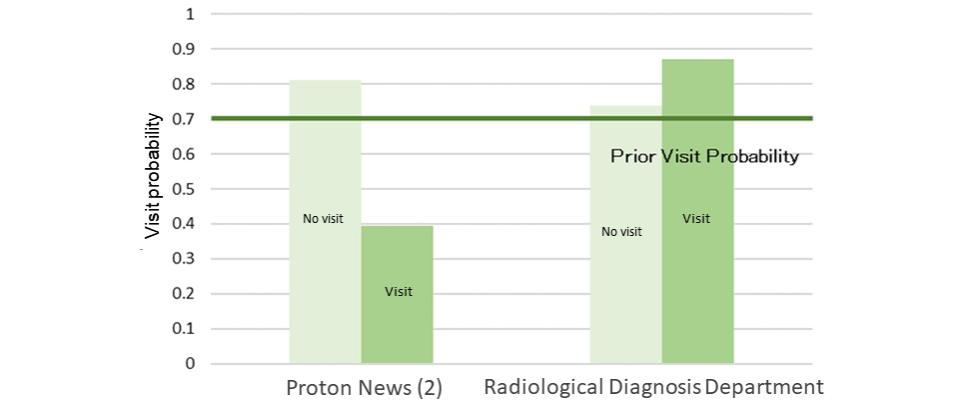
Probability inference (radiotherapy interest group, Radiotherapy Department page).

The model described in Figure 5 was structured using 35 sessions from the learning data. The browsing of RP2, the “Radiotherapy Department page,” was affected by the browsing of the Proton Therapy News page, which affected the browsing of the radiology-related department and Medical Expenses pages. The results of the sensitivity analysis are illustrated in Figure 6. The mutual information volume of the Proton Therapy News page was the largest, indicating that browsing this page had the greatest effect on the browsing of RP2. Based on the results of the sensitivity analysis, we calculated the change in the browsing probability of RP2 owing to browsing or nonbrowsing of the pages based on a probability inference, as shown in Figure 7. When the target visitor browses the Radiological Diagnosis Department page, the probability of RP2 increased by 17% from the prior probability.

### Nuclear Medicine Examination Interest Group

**Figure 8:**
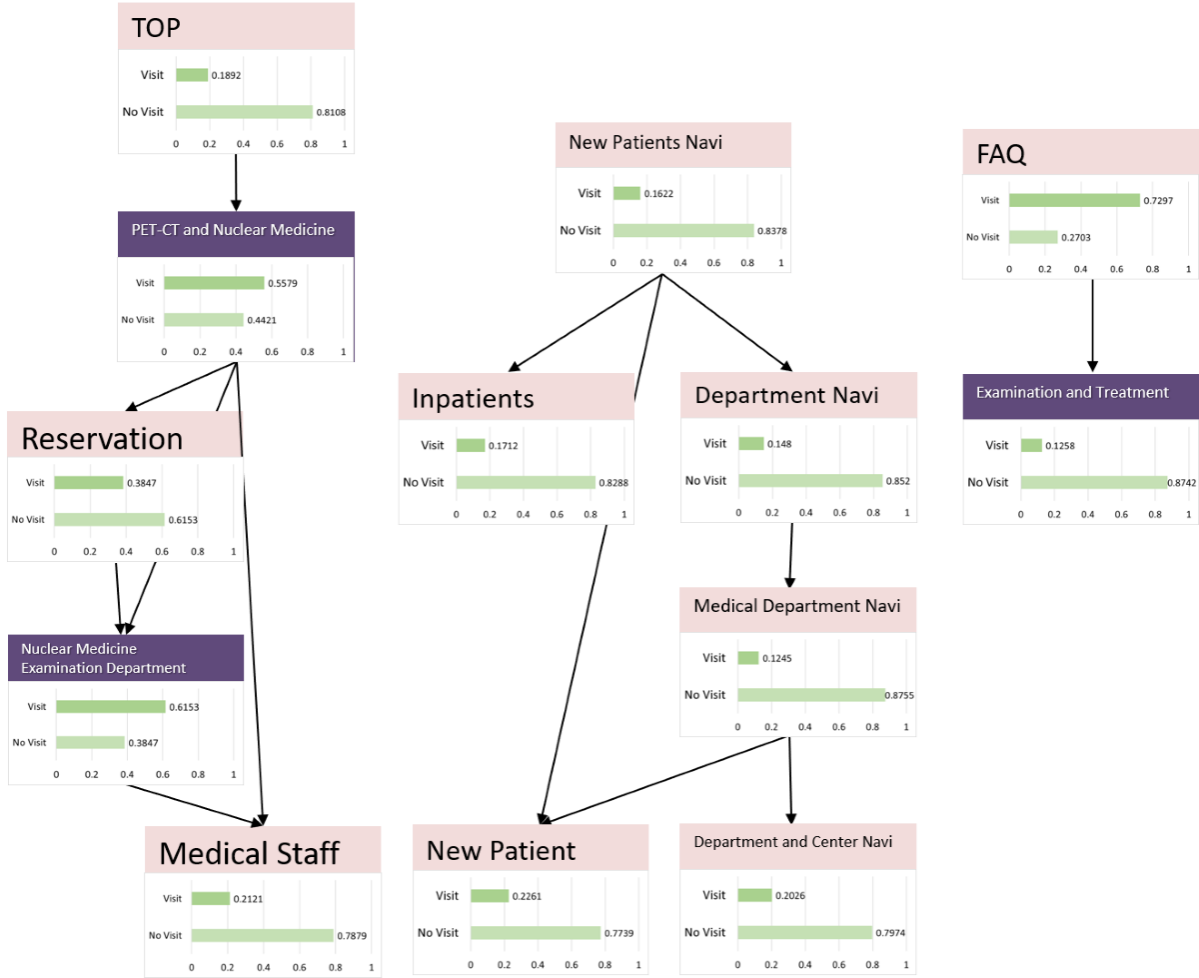
Nuclear medicine examination model.

**Figure 9:**
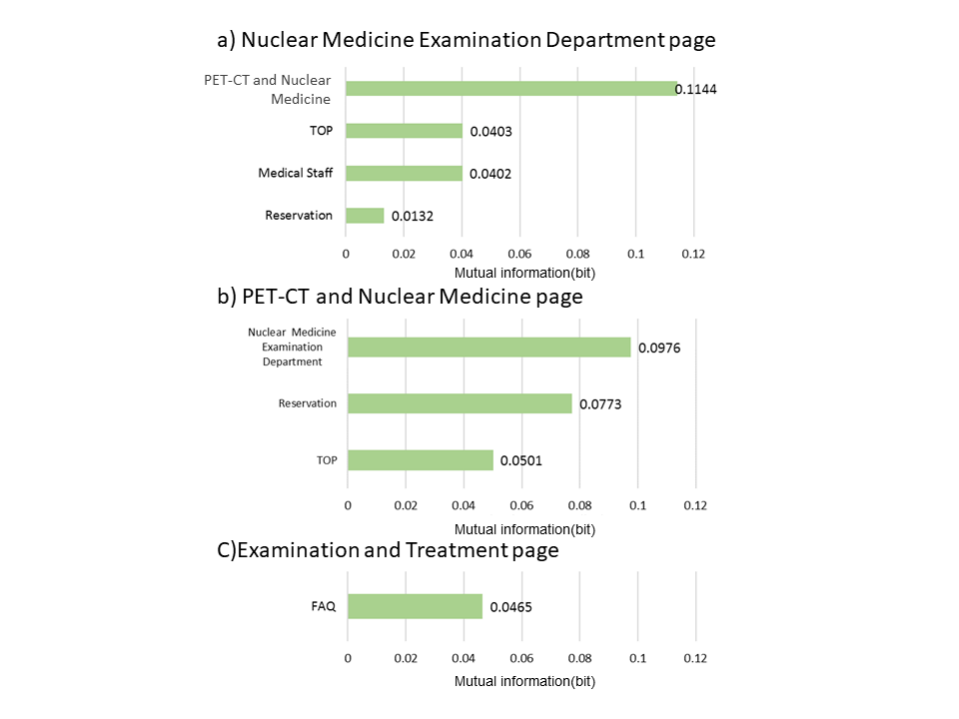
Sensitivity analysis (nuclear medicine examination interest group). PET-CT: positron emission tomography-computed tomography.

**Figure 10:**
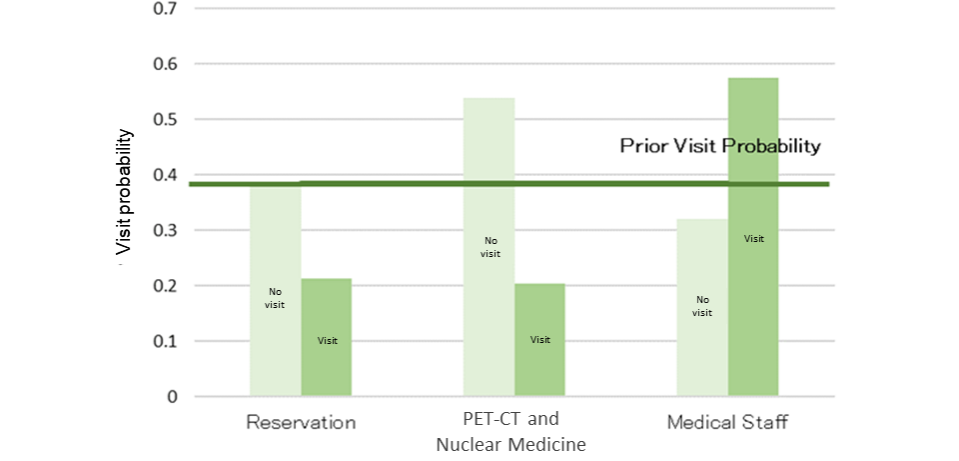
Probability inference (nuclear medicine examination interest group, Nuclear Medicine Examination Department page). PET-CT: positron emission tomography-computed tomography.

**Figure 11:**
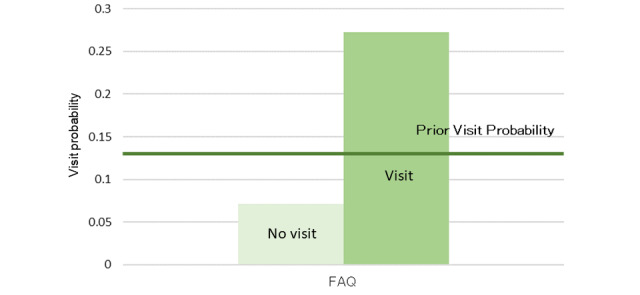
Probability inference (nuclear medicine examination interest group, Examination and Treatment page).

The model, as described in Figure 8, was structured using 35 sessions from the learning data. Browsing of the RP2 “Nuclear Medicine Department page” was affected by the browsing of RP4 “PRT-CT and Nuclear Medicine Examination” and was related to the browsing of the reservation and the medical staff pages. The results of the sensitivity analysis are shown in Figure 9. The interaction between the browsing of RP2 and that of RP4 was also demonstrated. We then calculated the change in the browsing probability of RP2 and RP3 owing to the browsing or nonbrowsing of the pages, as depicted in Figures 10 and 11. The browsing probability of RP2 increased when browsing the Medical Staff page. When the target visitors browsed the FAQ page, the browsing probability of RP3 increased by 15% from the prior probability.

### Radiological Diagnosis Interest Group

**Figure 12:**
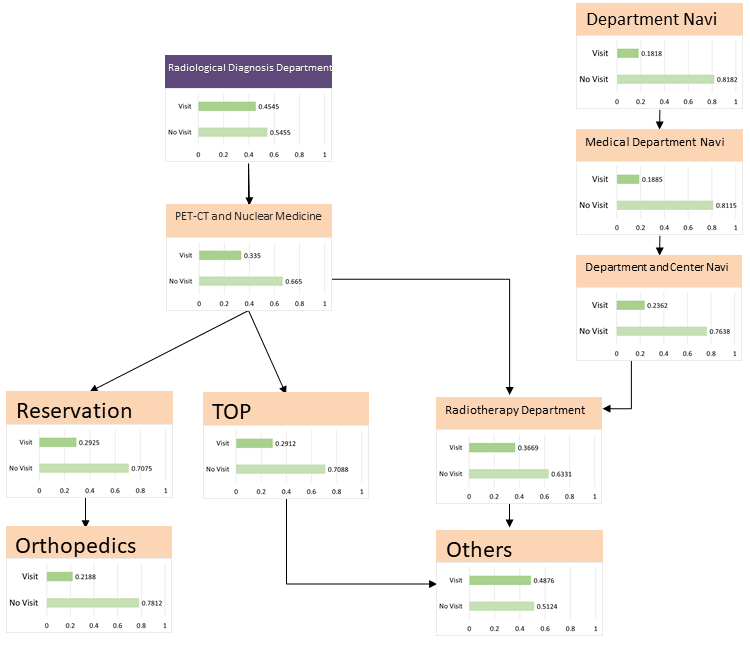
Radiological diagnosis model. PET-CT: positron emission tomography-computed tomography.

**Figure 13:**
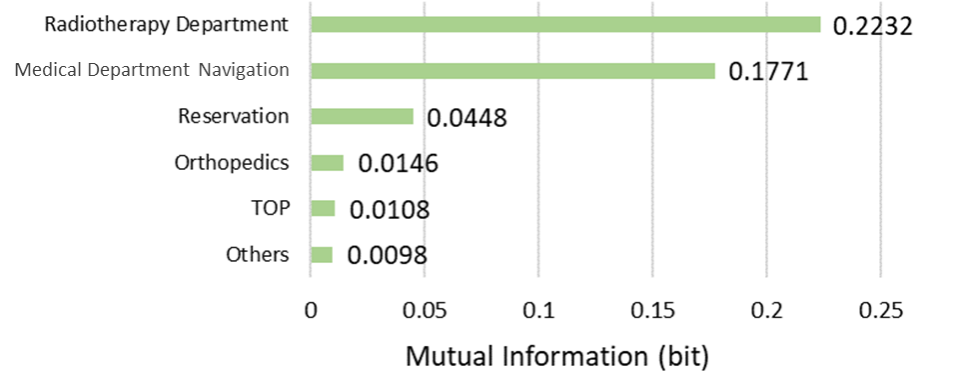
Sensitivity analysis (radiological diagnosis interest group).

**Figure 14:**
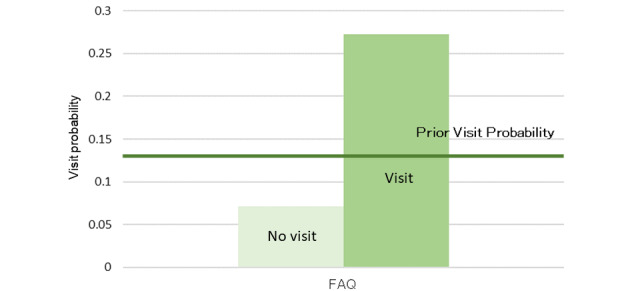
Probability inference (radiological diagnosis interest group, Radiological Diagnosis page).

The model described in Figure 12 was structured using 20 sessions from the learning data. The browsing of the RP2 “Radiological Diagnosis Department page” affected the browsing of the Radiotherapy Department and related department pages, such as Orthopedics. The results of the sensitivity analysis are shown in Figure 13. Browsing of the Radiotherapy Department page had the greatest impact on RP2 viewing. We then calculated the change in the browsing probability of RP2 owing to the browsing or nonbrowsing of the Radiotherapy Department page, as shown in Figure 14. When the target visitors browsed the Radiotherapy Department page, the probability of RP2 increased by 35% from the prior probability.

## Discussion

The number of visiting sessions on the Hokkaido University Hospital website is over 650,000 per year. This number is comparable to the number of actual patients to the hospital annually (approximately 740,000 in 2015) and indicates that the website plays a key role as an information provider for many individuals.

Only 2% (2/90) of the target visitors could reach the Radiology Center page (RP1), which describes its inspection information. There are 3 possible reasons for this finding: browsing behavior (the visitor started with the department pages, not the Center page), the visitor could not distinguish the Radiology Center pages from the radiology-related department pages that describe the department information, and that there are some pages for which we could not obtain the data access log. In total, 2 plans were considered for improving the browsing probability of RP1: linking the radiology-related department pages and the Radiology Center page, and summarizing radiology-related information on the website rather than posting according to the hospital’s organizational structure.

According to the radiotherapy model, when browsing the Proton Therapy News page, the browsing probability of the RP2 “Radiotherapy Department page” decreased. Therefore, we found that interest in radiotherapy and interest in news differed from one another, or that there was no conductor design. In addition, because the child node of the Radiotherapy Department page had the pages of new patients and medical expenses pages, patients who receive radiation treatment tend to seek information on receiving medical treatment such as medical expenses owing to the high price of proton therapy [26]. In a questionnaire survey of medical radiological specialists, it was reported that a certain number of technicians were asked about the cost of radiotherapy in treating patients receiving cancer radiotherapy [27].

In the nuclear medicine examination interest group, the browsing probability of the FAQ page was higher than that of the other 2 groups. In addition, as a result of the probability inference, a positive relationship was found between browsing the FAQ page and the Examination and Treatment page. Because the FAQ page is generally used for posting information on exceptional cases such as product guidelines and specific topics [28], target visitors may be concerned about specialized information, except for the content of the RP. From Figure 10, the browsing probability of the RP2 “Nuclear Medicine Examination Department page” was higher than the prior probability when browsing the Medical Staff page. Because neither of these pages were designed with a link that allows direct access, it is mainly the medical staff that browse them.

In the radiological diagnosis model, the RP2 “Radiological Diagnosis Department page” contained child nodes, such as the Orthopedics Department page, indicating that visitors are interested in the contents of the departments they are associated with. Table 6 presents the proportion of radiological diagnosis conducted at Hokkaido University Hospital, with the proportion of “bone/soft” being the second highest after “respiratory organs.” Therefore, the site design was considered, linking radiology-related department pages with their related department pages and redesigning the website considering the flow of treatment and inspection.

**Table 6:** Number of radiological examinations at Hokkaido University Hospital (April 2018 to March 2019).

Classification	Inspections, n (%)
Respiratory	53,322 (41.9)
Bone and soft part	30,230 (23.8)
Digestive organ	27,141 (21.3)
Tooth	16,685 (13.1)
Others	18 (0)

From the results of the probability inference, the browsing probability of the RP2 “Radiological Diagnosis Department page” was higher than the prior probability when browsing the Radiotherapy Department page. According to these results, visitors interested in radiology-related departments would access RP2, which indicates that CVR can be improved by linking radiology-related departments. The percentage of visitors who browsed the RP3 “Examination and Treatment page” was small. This is thought to be due to the low interest of the target visitor or their inability to reach the page. In the latter case, we suggest that CVR can be increased by linking to the “Radiology Department page,” where the targets have a high percentage of browsing.

Table 7 presents the site design proposals to increase the CVR, as examined from the results. Thus, this research concluded that our method is effective in improving the provision of information because improving the quality of decision making is crucial in data analytics, as exemplified through business intelligence tools [29]. The proposed method can be applied to departments other than radiology-related areas.

**Table 7:** Proposals for site design to meet the needs of visitors seeking radiology-related information.

RP^a^			Improvement 1		Improvement 2		Improvement 3
**A. Improve CVR^b^ of radiotherapy interest group**							
	RP1: Radiology Center page	Link the Radiology Center page and radiology-related department pages		Link the Radiology Center page and pages such as the New Patients page and Re-examination page		Set tracking code	
	RP2: Radiotherapy Department page	Link the Radiotherapy Department page and Radiological Diagnosis Department page		Link the Radiotherapy Department page and the Medical Expenses page		—^c^	
**B. Improve CVR of nuclear medicine examination interest group**							
	RP1: Radiology Center page	Same as above		Same as above		Same as above	
	RP2: Nuclear Medicine Examination Department page	Link the Nuclear Medicine Examination Department page and the Medical Staff page		—		—	
	RP3: Examination and Treatment page	Review of posted contents on the Examination and Treatment page		—		—	
	RP4: PRT-CT^d^ and Nuclear Medicine Examination page	Link the PRT-CT and Nuclear Medicine Examination page and the Nuclear Medicine Examination Department page		—		—	
**C. Improve CVR of radiological diagnosis interest group**							
	RP1: Radiology Center page	Same as above		Same as above		Same as above	
	RP2: Radiological Diagnosis Department page	Link Radiological Diagnosis Department page and the Radiotherapy Department page		Link the Radiological Diagnosis page and the Orthopedics page		—	
	RP3: Examination and Treatment page	Link the Radiological Diagnosis Department page and the Examination and Treatment page		—		—	

^a^RP: request page.

^b^CVR: conversion rate.

^c^The certain CVRs were treated as not applicable in our behavioral analysis.

^d^PRT-CT: positron emission tomography-computed tomography.

### Limitations

One of the limitations of this study is the low accuracy of the models applied. The number of samples (20-35 sessions) used to build the models was small compared with the actual usership for Hokkaido University Hospital’s website. Owing to the recent shift to Secure Sockets Layer for use in search engines, the sample size has remained limited. Future studies should substitute search keywords. Furthermore, some data may have been missing because there were some pages set with the no web tracking code in Google Analytics. By increasing the sample size, factors such as browsing time can be incorporated into the model, improving the prediction accuracy for the probability distribution of each page. However, the effects of the visitor’s interest on each page regarding factors such as the browsing time and browsing devices for hospital websites are still unknown. Therefore, it is necessary to clarify the effects of the browsing factors before modeling.

As another limitation, we were unable to verify the accuracy of the proposed model compared to other approaches. To evaluate our model, we need to accurately set the web tracking code and extract data without using retrieval keywords because we have obtained few retrieval data in recent years.

Third, the RP we set during the page-evaluation phase is uncertain. Thus, setting an RP objectively to obtain information by means of questionnaires to accurately understand visitor needs will be an issue in the future. Similarly, it will be possible to evaluate pages suitable for each visitor by clarifying the above relationship between visitor interests for each page and access indicators such as the browsing time. In recent years, Japan has been rapidly aging, and older adults are using hospital websites. However, previous studies have reported that age-related deterioration in visual acuity and changes in color vision affect the use of older adults to use such websites [30,31]. Therefore, we hope that the factors affecting CVR differ between older adults and young adults. In the future, we would like to consider improving the CVR by modeling the categorization of young and older adults.

### Conclusions

This study structured 3 browsing models, each based on specific patient needs: radiotherapy, nuclear medicine examination, and radiological diagnosis. A total of 74% (67/90) of the target visitors could reach their requested page, but only 2% (2/90) could reach the inspection information page owing to the site structure. Furthermore, for the radiological diagnosis interest group, browsing of the “Radiological Diagnosis Department page” affected the browsing of the related department pages, such as Orthopedics. 

Plans were considered for improving the browsing probability of the inspection information page such as summarizing radiology-related information on the website rather than posting according to the hospital’s organizational structure. Thus, our method has the potential to increase the probability of delivering the desired information to the users. In the future, we plan to improve the accuracy of the models by using a large number of samples. We believe that an increase in the number of subjects will reveal the characteristics of the browsing scenario in more detail.

We built models with the objective of improving the provisioning of radiology-related information; however, because the structure of a hospital website is not unique, we hope to apply the proposed method to other hospitals. It is expected that the provisioning of information on hospital websites will be improved using this approach.

## Appendix

Multimedia Appendix 1 Radiation-related search keyword.

## Abbreviations

CVR: conversion rate
RP: request page
