# Supplementary material for: A Bayesian Network–Based Browsing Model for Patients Seeking Radiology-Related Information on Hospital Websites: Development and Usability Study
Source: J Med Internet Res. 2021 Jan 19;23(1):e14794. doi: 10.2196/14794 (PMC7854043; doi:10.2196/14794)
Supplement: Multimedia Appendix 1 [file jmir_v23i1e14794_app1.pdf]

Appendices. Radiation-related search keyword.

Keyword
Radiology
Radioactivity
General Shooting
CT
MRI
Porter
Linac
Angiography
Dose
Mammography
Proton Beam
X-rays
Molecular Imaging
Exposure
Radiology Source
Bone Density Test
Treatment Plan
TV inspection
SPECT
Angiography
Nuclear Medicine

Cyclotron
Isotope
Scintigraphy
DEXA (Dual Energy X-ray Absorptiometry)
PET (Positron Emission Tomography)
Fluoroscopy
IVR (Interventional Radiology)
DSA (Digital Subtraction Angiography)
Moving Body Tracing Radiology Device
FPD (Flat Panel Detector)
IMRT<br><br>(Intensity Modulated Radiation Therapy)
